# The impact of a radiologist‐led workshop on MRI target volume delineation for radiotherapy

**DOI:** 10.1002/jmrs.298

**Published:** 2018-08-03

**Authors:** Shivani Kumar, Lois Holloway, Dale Roach, Elise Pogson, Jacqueline Veera, Vikneswary Batumalai, Karen Lim, Geoff P. Delaney, Elizabeth Lazarus, Nira Borok, Daniel Moses, Michael G. Jameson, Shalini Vinod

**Affiliations:** ^1^ South Western Sydney Clinical School Faculty of Medicine University of New South Wales Sydney New South Wales Australia; ^2^ Liverpool and Macarthur Cancer Therapy Centres Liverpool Hospital Liverpool New South Wales Australia; ^3^ Ingham Institute of Applied Medical Research Liverpool New South Wales Australia; ^4^ Centre for Medical Radiation Physics University of Wollongong Sydney New South Wales Australia; ^5^ Institute of Medical Physics School of Physics University of Sydney Sydney New South Wales Australia; ^6^ Peter MacCallum Cancer Centre Bendigo Victoria Australia; ^7^ University of Western Sydney Sydney New South Wales Australia; ^8^ Department of Radiology Liverpool Hospital Liverpool New South Wales Australia; ^9^ Department of Radiology Prince of Wales Hospital Randwick New South Wales Australia; ^10^ Prince of Wales Clinical School Faculty of Medicine University of New South Wales Sydney New South Wales Australia

**Keywords:** Education, inter‐observer variability, MRI, target volume delineation, training

## Abstract

**Introduction:**

Magnetic resonance imaging (MRI) is increasingly used for target volume delineation in radiotherapy due to its superior soft tissue visualisation compared to computed tomography (CT). The aim of this study was to assess the impact of a radiologist‐led workshop on inter‐observer variability in volume delineation on MRI.

**Methods:**

Data from three separate studies evaluating the impact of MRI in lung, breast and cervix were collated. At pre‐workshop evaluation, observers involved in each clinical site were instructed to delineate specified volumes. Radiologists specialising in each cancer site conducted an interactive workshop on interpretation of images and anatomy for each clinical site. At post‐workshop evaluation, observers repeated delineation a minimum of 2 weeks after the workshops. Inter‐observer variability was evaluated using dice similarity coefficient (DSC) and volume similarity (VOLSIM) index comparing reference and observer volumes.

**Results:**

Post‐workshop primary gross tumour volumes (GTV) were smaller than pre‐workshop volumes for lung with a mean percentage reduction of 10.4%. Breast clinical target volumes (CTV) were similar but seroma volumes were smaller post‐workshop on both supine (65% reduction) and prone MRI (73% reduction). Based on DSC scores, improvement in inter‐observer variability was seen for the seroma cavity volume on prone MRI with a reduction in DSC score range from 0.4–0.8 to 0.7–0.9. Breast CTV demonstrated good inter‐observer variability scores (mean DSC 0.9) for both pre‐ and post‐workshop. Post‐workshop observer delineated cervix GTV was smaller than pre‐workshop by 26.9%.

**Conclusion:**

A radiologist‐led workshop did not significantly reduce inter‐observer variability in volume delineation for the three clinical sites. However, some improvement was noted in delineation of breast CTV, seroma volumes and cervix GTV.

## Introduction

Accurate and reproducible delineation of target volumes and organs at risk (OARs) is a prerequisite for effective high‐dose conformal radiation therapy. Variability in volume delineation is the largest source of error in radiotherapy.^1^ Multi‐modality imaging can be utilised to improve visualisation and minimise this error.^1^, ^2^ Pathological tumour boundaries remain the gold standard for assessing accuracy of observer volume delineation on different imaging modalities, however, data on this is limited.^3^, ^4^, ^5^, ^6^ A surrogate measure is the agreement of tumour volumes delineated by multiple observers on different imaging modalities.^2^

Magnetic resonance imaging (MRI) is increasingly being utilised in radiotherapy for gross target volume (GTV) and OAR delineation.^7^ MRI has the advantage of superior soft tissue contrast making it a superior imaging modality compared to computed tomography (CT) images for volume delineation. However, anatomical appearances differ between CT and MRI. CT allows differentiation of tissues that border air, bone or fat, however, image intensity of surrounding soft tissues remains relatively constant. Therefore, CT displays poor contrast resolution between surrounding normal tissue and tumour boundaries. On MRI, image intensity of different body tissues can vary depending on the type of MRI sequence. For example, on a T1‐weighted image, fat appears brighter and water appears darker while on T2 images water appears brighter and fat shows varying levels of intensity.

For treatment sites such as brain^8^, ^9^ and prostate,^10^, ^11^, ^12^ MRI is increasingly used for delineation of GTV and OARs. Reduction in inter‐observer variability has been demonstrated with the incorporation of MRI into the radiotherapy workflow.^12^, ^13^, ^14^ Despite the increased availability and utilisation of MRI data to facilitate volume delineation, there are few consensus guidelines and atlases, as exists for CT‐based volume delineation.^15^, ^16^ Guidelines improve consistency in contouring which can lead to an improvement in precision in radiotherapy.^16^, ^17^, ^18^ This becomes important with the introduction of a new imaging modality into the radiotherapy workflow.^19^

There have been a limited number of studies which have demonstrated reduction in inter‐observer variability in volume delineation with the introduction of an educational programme or workshop.^20^, ^21^, ^22^ With the rapid uptake of MRI in radiotherapy planning workflow, the aim of this study was to evaluate whether a MRI workshop led by a radiologist reduced inter‐observer variability in target volume delineation for three different clinical sites.

## Methodology

### Study cases

Data from three separate studies assessing the impact of MRI in radiotherapy planning for lung, breast and cervix cancers were selected. Each study had a component that assessed target volume delineation before and after a radiologist‐led education workshop. These studies were being performed to educate observers before larger planned studies evaluating the impact of MRI on target volume delineation for the same sites. These studies were approved by the institutional Human Research Ethics Committee. For the lung study, three patients (Patient 1: T2N2M0, Patient 2: T2N1M0 & Patient 3: T2N2M0 non‐small cell lung cancer) were selected, with four radiation oncology observers and one thoracic radiologist observer from three different tertiary hospitals. There was one post‐operative breast cancer case (T1bN0M0) with six observers (four radiation oncologists and two radiologists) from a single centre. The cervix cancer study included one patient case (T2bN0M0) with eight observers (six radiation oncologist and two radiologists) from six tertiary hospitals. All observers participating in the study had more than 5 years of clinical experience working in their sub‐specialities.

### Imaging

Lung images were acquired on a 1.5T MRI scanner with the surface coil placed directly on the patient's thorax to maximise signal. Patients were positioned in a supine position with their arms above their head. The field of view encompassed the entire thorax, with a 20‐sec breath‐hold instruction at inspiration given to patients during imaging. Breast images were acquired on a 3T scanner, patients underwent scans in two positions: (1) supine with a vacbag on a flat wing board (MTWB09 Wingboard; CIVCO Medical Solutions, Orange City, IA) with arms raised above the head; and (2) prone position, on a dedicated breast coil (Sentinelle Breast MRI System, Hologic, Bedford, MA). The supine MRI was acquired with a surface coil held close to but not touching the breast tissue (to avoid deformation of the anterior breast contour) using a foam bridge. Cervix images were acquired on a 1.5T scanner in a supine position, with surface coil placed directly on patient pelvis. Detailed imaging parameters are presented in Table 1.

**Table 1:** Imaging parameters for all three clinical sites

MRI acquisition parameters	Clinical sites		
	Lung	Breast	Cervix
Scanner	1.5T (Ge Signa HDE) Gradient strength 23mT/m	3T (Siemens Medical Systems, Erlangen, Germany)	1.5T (Symphony, Siemens medical Systems, Erlangen Germany) 24mT/m
Receiver coils	8‐channel surface coil	Supine : 18‐channel surface coil Prone: 16‐channel breast coil	6‐channel surface coil
Imaging sequence	T2: SSFSE T1: LAVA	T2: TSE	T2: 3D TSE (SPACE)
Acquisition plane	Transverse	Transverse	Transverse
Slice thickness	6 mm	2 mm	2.5 mm

SSFSE, single‐shot fast‐spin echo; LAVA, Liver acquisition with volume acceleration; TSE, turbo‐spin echo; SPACE, sampling perfection with application optimised contrast.

### Pre‐workshop volume delineation

All observers were asked to delineate baseline target volumes prior to the workshop. Observers were given clinical and imaging information pertinent to the clinical site. Observers delineating on the lung datasets were provided with each patient's clinical history, diagnostic CT and planning PET report. Observers were instructed to delineate the primary and nodal GTV. The planning CT along with the T1‐ and T2‐weighted MRI image dataset was provided for delineation. For the breast case, no additional patient‐specific imaging report or patient history was provided, all observers were instructed to delineate the whole breast tissue as clinical target volume (CTV) and the seroma cavity on the supine and prone T2‐weighted MRI provided. Observers for the cervix case study were instructed to contour the GTV and specific structures (uterus, cervix, vagina and parametrium) individually and then combine these structures to create the CTV. To assist in volume delineation for cervix cases, observers were given the diagnostic MRI and PET report as well as examination under anaesthesia report. Observers were given the planning CT and the T2‐weighted MRI dataset for delineation.

### Radiologist led workshop

Site‐specific training in target volume delineation was given by expert radiologists sub‐specialised in each clinical site. Each clinical site workshop was conducted separately as a group session lasting for 2 h. The workshops were interactive with the expert radiologist discussing interpretation of MRI images and definition of tumour volumes with the observers.

### Post‐workshop volume delineation

After a minimum of a 2 weeks gap, the observers were asked to delineate their post‐workshop target volumes on the datasets. The initial instructions were again provided to the clinicians to assist with delineation and an additional MRI‐based contouring guide specific to each site was also provided.

### Reference volume

Each clinical site selected a different strategy to define the reference volume to reflect the true volume. For lung cases, the radiologist volume was selected as the reference volume, as it was felt that a thoracic radiologist volume would most closely represent the true volume given their experience in thoracic MRI compared to radiation oncologists. Simultaneous truth and performance level estimation (STAPLE)^23^, ^24^ volume based on pre‐ and post‐workshop breast contours was used as the reference volumes for pre‐ and post‐workshop contour analysis for the breast dataset. This was performed to directly compare inter‐observer variation without bias towards pre‐ or post‐workshop datasets and variation in STAPLE volume. A 90% confidence level for agreement was used for STAPLE generation within CERR (a computational environment for radiation therapy research) software within the MATLAB R2012a platform (MathWorks, Natick, MA). The reference volume for the cervix dataset was generated based on expert clinician consensus. The expert clinician consensus contour was determined by a group review of each structure by four experienced radiation oncologists and one radiologist and a consensus contoured agreed on by all clinicians.

### Analysis of delineation uncertainties

**Figure 1:**
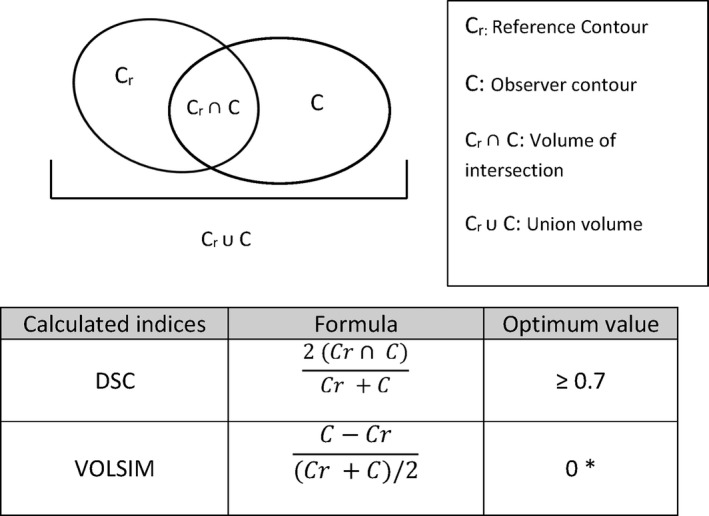
Definition of contour analysis metrics. * A VOLSIM value of > 0 indicates observer volume is larger than reference volume and < 0 indicated observe volume is smaller than reference volume. This index does not take into account any overlap measure.

Deviations of the observer volumes from the reference volume were assessed using open‐source image manipulation software MilxView.^25^ Dice similarity coefficient (DSC) and volume similarity (VOLSIM) were calculated (Fig. 1). Dice similarity coefficient was used as measure of overlap to assess inter‐observer variability.^26^ A DSC ≥ 0.7 was considered ‘good’ overlap between reference and observer volume.^2^ VOLSIM is a volume‐based metric that considers the absolute volume of the segmented region compared with another segmented region, it does not take into account the overlap of volumes.^27^

## Results

### Volume comparison

#### Lung

**Figure 2:**
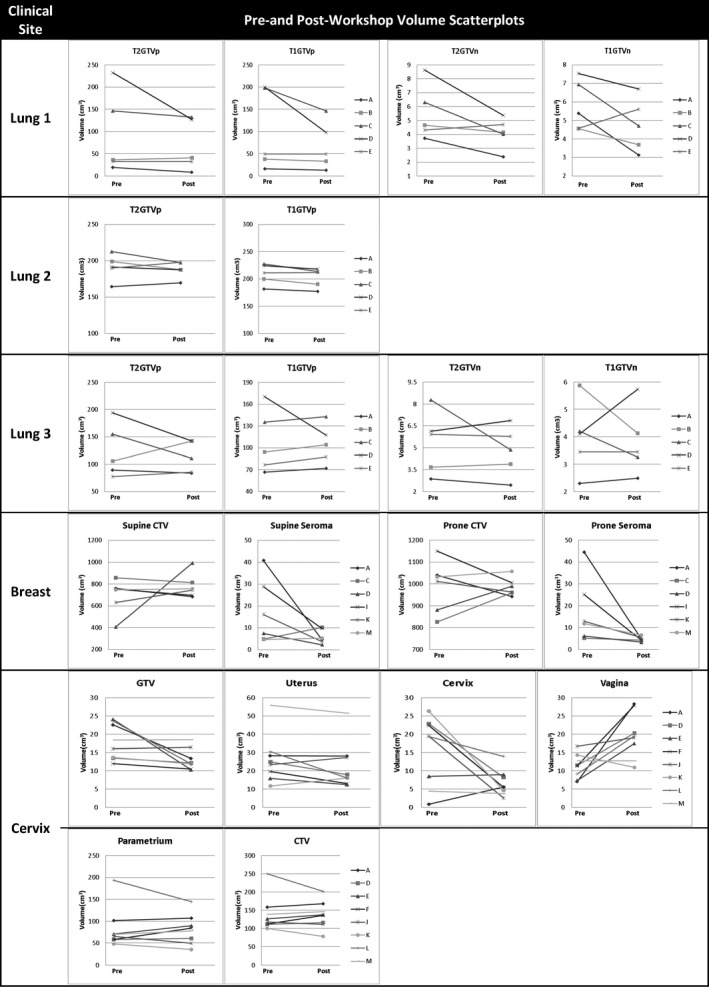
Individual observer contour volumes for each clinical site and volume delineated pre‐ and post‐workshop. T2GTVp, T2‐weighted primary gross tumour volume; T1GTVp, T1‐weighted primary gross tumour volume; T2GTVn, T2‐weighted nodal gross tumour volume; T1GTVn, T1‐weighted nodal gross tumour volume; CTV, clinical target volume; Pre, pre‐workshop volume; post, post‐workshop volume.

**Figure 3:**
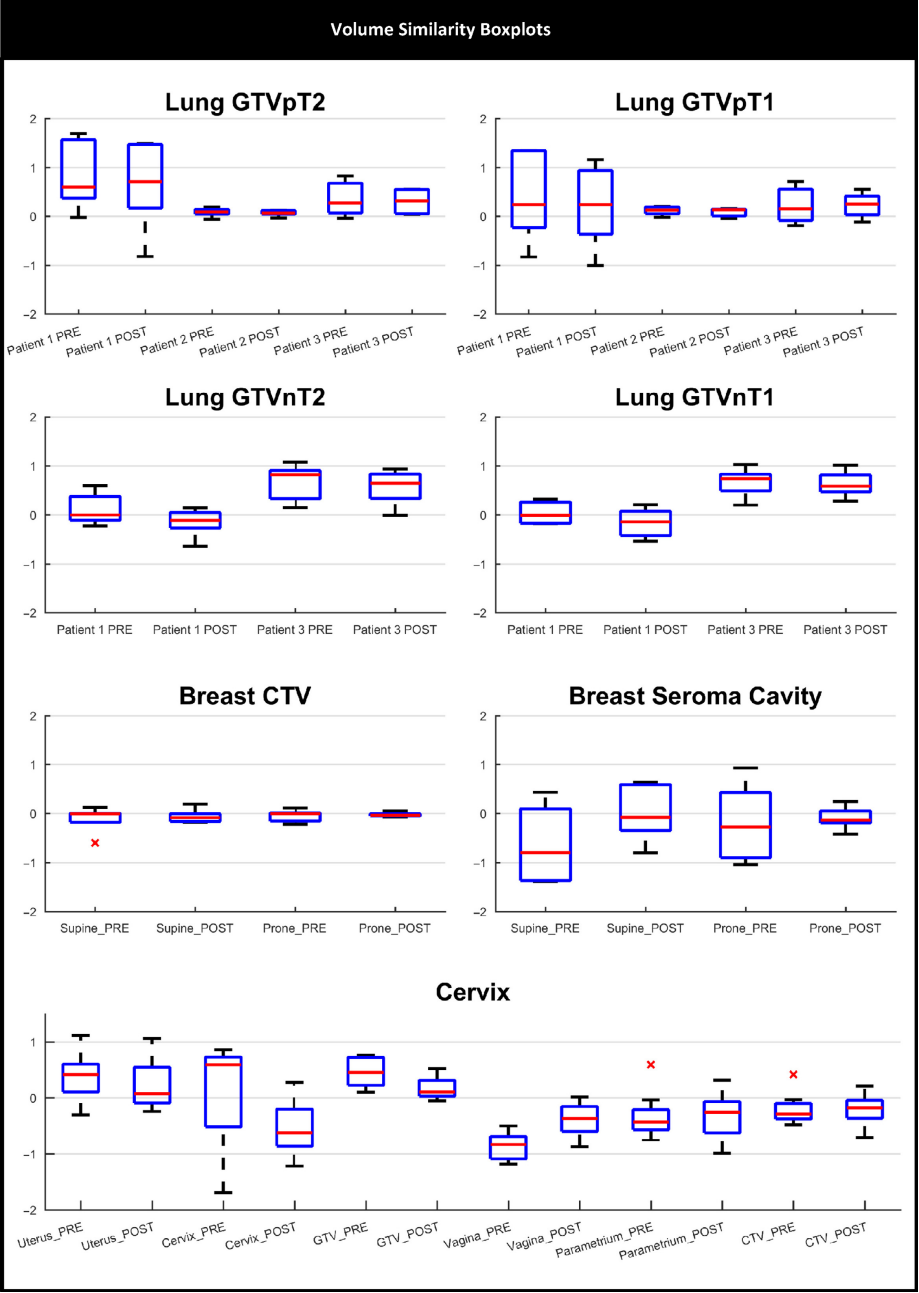
Volume similarity boxplot for all clinical sites. *Indicates outlier observer scores. VOLSIM, volume similarity.

Lung patients 1 and 3 demonstrated observer variation in defining the boundary between primary GTV (GTVp) and atelectasis (Appendix I). Post‐workshop GTVps for most observers were marginally smaller compared to pre‐workshop volumes (Fig. 2). For GTVp pre‐ and post‐workshop volumes, mean absolute difference was 10.4% (range 0–32%). Patient 1 demonstrated the largest mean difference between pre‐ and post‐workshop volumes (32%). No differences were noted between T1‐ and T2‐weighted volumes (Fig. 2). Patient 2 had N2 nodal disease stage within close proximity to the primary disease and thus the nodal disease volume was incorporated into the primary GTV. Compared to the reference volume (disregarding overlap measure) there was large spread in VOLSIM scores for GTVp and GTVn for patients 1 and 3 (Fig. 3). Patient 2 GTVp was similar in volume to reference observer (Fig. 3).

#### Breast

Post‐workshop supine and prone seroma cavity volumes were smaller compared to pre‐workshop volumes for almost all observers; breast CTV did not demonstrate a trend between pre‐ and post‐workshop volumes. (Fig. 2). Mean CTV increased post‐workshop on the supine dataset by 12.8%, with minimal change noted on prone MRI. Average seroma cavity volume reduced by 65 and 72.8%, respectively, for supine and prone images post‐workshop. Prone post‐workshop CTV and seroma cavity volumes demonstrated the least variation among observer VOLSIM scores, with observer volumes being predominantly smaller (VOLSIM < 0) compared to reference STAPLE pre‐ and post‐workshop volumes (Fig. 3). Variations in majority of the breast contours are shown in Appendix I.

#### Cervix

On average GTV volume reduced by 26.9%, cervix by 57.2% and vagina volume increased by 73% between pre‐ and post‐workshop. The CTV was similar pre‐ and post‐workshop (mean volume of 139.2 and 136.8 cc pre‐ and post‐workshop, respectively). Outlier observers were noted for uterus, parametrium and CTV (Fig. 2). Compared to reference volume, uterus and GTV observer volumes were larger pre‐ and post‐workshop (VOLSIM > 0) (Fig. 3). Large observer variation pre‐ and post‐workshop in defining posterior uterus border and superior inferior extent of vaginal contours (Appendix I) was noted. Cervix contour demonstrated a large reduction in inferior margin, while parametrium volume remained variable both pre‐ and post‐workshop.

### Inter‐observer variability assessment

#### Lung

**Figure 4:**
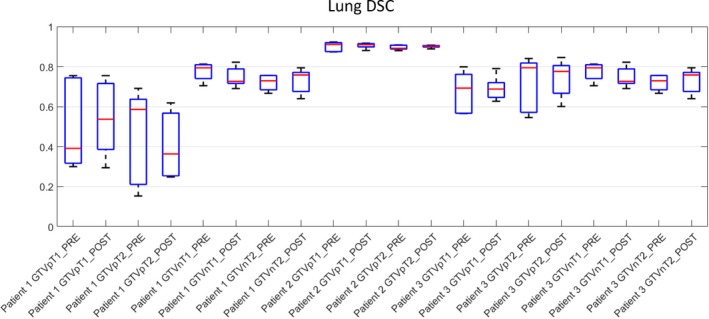
Lung DSC boxplots of the three lung cases for all observers. DSC, dice similarity coefficient.

Variation in DSC scores was seen between the three lung cases (Fig. 4). The average DSC score did not differ pre‐ and post‐workshop (mean percentage difference was 0%). Patient 1 had large variation in inter‐observer variability as can be seen by the spread of observer DSC scores (Fig. 4). The DSC scores ranged from 0.30 to 0.75 for GTVp on T1 images to 0.15 to 0.9 on T2 images (Fig. 4). Patients 1 and 3 showed a reduction in the spread of DSC scores in the delineation of primary GTV volumes post‐workshop. Patient 2 demonstrated good agreement in volume delineation both pre‐ and post‐workshop volumes for primary and nodal disease.

#### Breast

**Figure 5:**
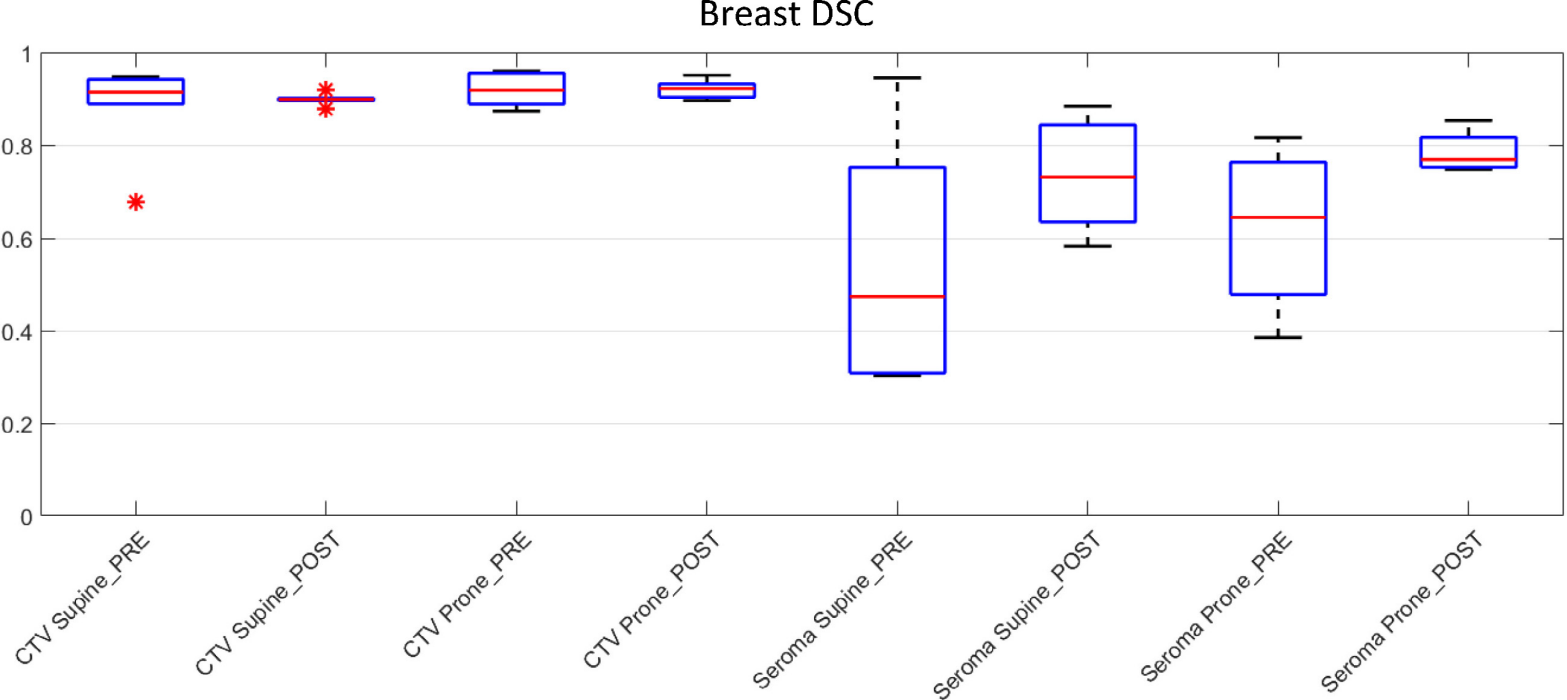
Breast DSC boxplots for all observers. *Indicates outlier observer score. DCS, dice similarity coefficient; CTV, clinical target volume.

Breast CTV showed good initial inter‐observer agreement in volume delineation for both supine and prone positions, range 0.95–0.68 for supine and 0.96–0.88 for prone DSC score improvement was noted for both positions post‐workshop (Fig. 5). The largest spread of observer scores was seen in the pre‐workshop seroma cavity volumes on supine (DSC 0.3–0.9) and prone (DSC 0.4–0.8) MRI (Fig. 5). The greatest improvement in inter‐observer agreement was noted for seroma cavity volumes on the prone MRI, with DSC range improving from 0.4–0.8 pre‐workshop to 0.7–0.9 post‐workshop.

#### Cervix

**Figure 6:**
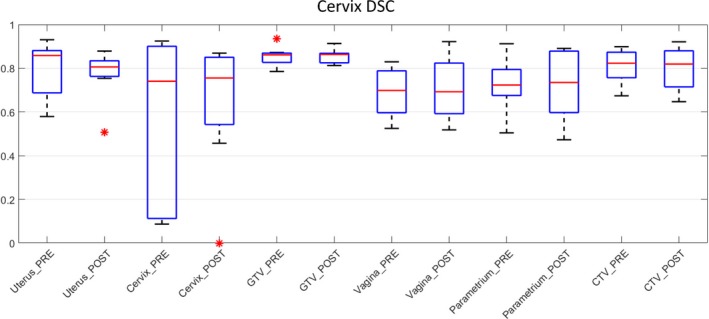
Cervix DSC boxplot for all observers. *Indicates outlier observer score. DCS, dice similarity coefficient; GTV, gross tumour volumes; CTV, clinical target volume.

GTV, uterus and cervix volumes for the cervix data showed improved agreement post‐workshop based on the spread of the DSC scores (Fig. 6). Uterus and cervix volumes demonstrate outlier observer DSC scores of 0.5 and 0 respectively. Vagina, parametrium and CTV showed poorer observer agreement with the reference volume post‐workshop, with larger spread in DSC scores.

## Discussion

Volume delineation in radiotherapy planning is a complex task and requires guidelines to improve accuracy and consistency.^28^ Contour variation can exceed geometric error^29^ and have an impact on treatment outcome.^1^ The objective of this study was to investigate the impact of teaching interventions for lung, breast and cervix cancer MRI‐based delineation. The clinical sites chosen are on a spectrum of MRI utility for delineation of radiotherapy volumes. MRI is well established as an imaging modality for improved target volume delineation in cervix cancer.^30^Breast MRI is established for diagnosis of breast cancers, especially in cases not seen on mammogram or ultrasound, however, it is not routinely used for breast radiotherapy.^31^ The use of MRI in lung cancer is largely confined to Pancoast tumours which infiltrate the brachial plexus and/or spinal canal. It is not used to image other lung cancers and its use is purely investigational. However, in the era of MRI linac development, there is renewed interest in developing MRI protocols for lung cancer radiotherapy, hence the motivation to include it in this study.

The routine imaging modality used to train radiation oncologists is CT imaging. Training in interpretation and use of MRI is variable depending on access. We therefore assessed whether a radiologist‐led workshop would improve target volume delineation for the three chosen clinical sites. Our findings were not consistent with only some volumes showing reduction in inter‐observer variability after the workshop. The largest improvement was seen in seroma cavity delineation, in the prone setup position for breast.

These results slightly differ from other studies which have demonstrated the impact of a contouring workshop or teaching session in improving inter‐observer variability.^13^, ^20^, ^21^, ^32^, ^33^ Of these, only two studies assessed an education intervention on MRI data and these were both for prostate treatment sites.^20^, ^21^ Delineating prostate tumour volumes on MRI is a well‐established radiotherapy practice and prostate anatomy does not vary significantly between patients unlike lung and cervix cancers. Previous studies that evaluated an educational intervention for lung tumour delineation were based on CT data only.^32^, ^33^ There are well‐established protocols for CT‐based contouring with and without positron emission tomography data for lung as this is standard practice. This is one of the few study to evaluate MRI contouring for lung cancers.

This study compared the impact of an educational MRI workshop for different clinical sites, where MRI is and is not routinely used for target volume delineation. For lung volumes, patient‐specific factors had a larger effect on inter‐observer variability based on DSC score. Patients 1 and 3 who had tumours with surrounding atelectasis demonstrated the largest variation in tumour boundary definition. Tumour and atelectasis interface variability between observers was also demonstrated by Karki et al.^34^ For some observers, there was minimal difference between their pre‐ and post‐ workshop volumes suggesting lack of an effect of education. For cervix and breast tumour sites where MRI is utilised in the diagnostic setting, and particularly cervix where MRI is used routinely for brachytherapy, only slight improvements in volume delineation was noted for selected contours. Pre‐workshop low inter‐observer variability scores may indicate that inclusion of MRI data alone do not improve contouring variability.

The results from this study highlight the challenge of introducing MRI into the radiotherapy planning process. The inclusion of the radiologist‐led workshop did not show a significant impact on improving inter‐observer variability for all sites and structures; however, a pattern in reduction in DSC scores for seroma cavity, breast CTV, cervix, cervix GTV and uterus was seen. With the rapid uptake of MRI into radiotherapy planning process this needs to be taken into account.

There are limitations to this study. The data was obtained from pilot studies in three clinical sites, with limited sample size and slightly differing methodology. Selection of the reference volume between each clinical site was different. While the radiologist volume would have been ideal reference contour across all three sites, differences in GTV delineation between radiation oncologists and radiologist have been reported^35^ and a more collaborative approach to volume delineation is suggested as the ideal approach.^36^ The clinical information provided for volume delineation also varied according to what was clinically relevant for each site. For lung GTV delineation, PET images were omitted to allow evaluation of volume delineation on MRI alone so as not to confound the contouring results based on interpretation of a second imaging modality. For breast no additional clinical information was given, to ensure the study evaluated the utility of the images alone as the target volume was the whole breast.

The teaching method was performed in an informal setting and the study did not assess whether the teaching intervention had a lasting effect on the observers to influence their volume delineation outside of the study. Changes in clinician confidence for volume delineation before and after the workshop was not measured. Variation in clinician experience with utilising MRI data was also not assessed, which may have an effect on improving delineation agreement at an individual clinician level. Technical factors such as MRI slice thickness and resolution may have had an impact on delineation variability. While MRI has excellent soft tissue resolution, motion artefacts can impact image resolution and thus contouring variability. It should also be noted that all imaging was performed without contrast injection to highlight nodal disease, tumour volumes or seroma cavity.

## Conclusion

A radiologist‐led workshop did not significantly reduce inter‐observer variability in volume delineation for the three clinical sites studied, except for seroma cavity volumes and selected cervix contours. As MRI is increasingly being adopted into radiotherapy planning and treatment, appropriate training and education needs should be considered to allow for this change in practice, particularly for sites currently not routinely using MRI. Further research is needed to design the most appropriate education or training intervention to support the use of MRI in radiotherapy.

## Conflict of Interest

The authors have no conflict of interest to declare.
